# Universal healthcare coverage, patients' rights, and nurse-patient communication: a critical review of the evidence

**DOI:** 10.1186/s12912-022-00833-1

**Published:** 2022-03-07

**Authors:** Abukari Kwame, Pammla M. Petrucka

**Affiliations:** 1 College of Graduate and Postdoctoral Studies, University of Saskatchewan, Saskatoon, Canada; 2 College of Nursing, University of Saskatchewan, Regina Campus, Regina, Canada

**Keywords:** Universal healthcare access, Patient-rights, The right to health, Sustainable development goals, Social determinants of health

## Abstract

The Sustainable Development Goals adopted by world leaders on September 25, 2015, aimed to end poverty and hunger, promote gender equity, empower women and girls, and ensure human dignity and equality by all human beings in a healthy environment. These development goals were premised on international human rights norms and institutions, thereby acknowledging the relevance of human rights in achieving each goal. Particularly, sustainable development goal 3, whose objective is to achieve universal health coverage, enhance healthy lives, and promote well-being for all, implicitly recognizes the right to health as crucial. Our focus in this paper is to discuss how promoting patients’ rights and enhancing effective nurse-patient communication in the healthcare setting is a significant and necessary way to achieve universal health coverage. Through a critical review of the empirical research evidence, we demonstrated that enhancing patients’ rights and effect nurse-patient communication will promote people-centered care, improve patients’ satisfaction of care outcomes, increase utilization of care services, and empower individuals and families to self-advocate for their health. These steps directly impact primary healthcare strategies and the social determinants of health as core components to achieving universal health coverage. We argue that without paying attention to the human rights dimensions or employing human rights strategies, implementing the other efforts will be inadequate and unsustainable in protecting the poorest and most vulnerable populations in the achievement of goal 3.

## Background

On September 25, 2015, world leaders at the United Nations (UN) General Assembly adopted a set of 17 sustainable development goals (SDGs) and 69 targets in an agenda titled *Transforming our World: The 2030 Agenda for Sustainable Development,* in which global leaders pledged to take bold and transformative steps to free the world and all people from poverty and hunger, to promote gender equity and empower women and girls, and to ensure that human dignity and equality are enjoyed by all human beings in a healthy environment [1]. These far-reaching, people-centered, and transformative sets of goals and targets focused on ensuring that “no one is left behind” as the world forges ahead to achieve economic, social, and environmental sustainability in its development programs, practices, and policies by 2030.

Recognizing the values of human dignity and equality and the impact development programs, practices, and policies have on these values, the SDGs were anchored on international human rights law, norms, and provisions, as emphasized in the Preamble, the Declaration, and other sections of the agenda document. In particular, the significance of health as a human right and the need for universal access to health by all was captured in SDG3, whose focus is to "ensure healthy lives and promote well-being for all, at all ages." [1, p. 14] Yet, despite recognizing the right to health in SDG3 and the significance of implementing the SGDs with attention to human rights principles as emphasized in the SDGs Agenda, the right to health is only implicitly referenced in targets 3.7 and 3.8 of SDG3.

Without much attention to human rights and the right to health principles in the universal health coverage (UHC) discourse or strategies to implement the targets, the approaches outlined in SDG3 will be inadequate to protect the poorest and most vulnerable populations in the achievement of SDG3 [2, 3]. Implementing UHC ensures that all people can obtain the health services they desire without suffering significant financial hardship when accessing those services [4]. For UHC to thrive, there must be equity in access to care services so that all those who need these services can get them. In addition, care services must be of high quality to improve the health of the people receiving them, and accessing the services must guarantee financial-risk protection such that the cost of using the service does not put undue financial stress on the users [4, 5].

Moreover, as a right to health issue, UHC recognizes the importance of the social determinants of health (SDH), the role of primary healthcare, and the impact of both internal and external healthcare institutional factors [5–8]. Furthermore, healthcare issues, such as coverage vs. access, access vs. quality, and care itself, are significant in the UHC discourses. These issues need to be critically examined, as Fried et al. [5] argued that access to healthcare services does not often guarantee quality services or satisfaction of care outcomes. We explore the question: How does a consideration of patient rights and the right to health contribute to achieving SDG3 and the global discourse of universal health coverage? Thus, we aim to present and discuss the UHC of SDG3 from a rights-based approach. Further, we will examine how effective nurse-patient communication and respect for patients' rights are crucial to achieving UHC; hence, emphasizing the right to health and patients' rights in the UHC discourse is desired now than ever. To achieve this paper's objectives, we will first discuss UHC as a human rights issue. Next, we examine the right to health and patients' rights, effective nurse-patient communication, and patient-centered care as crucial to achieving UHC. We then present a position statement for consideration in health policy, decision-making, and practice before concluding the paper.

Critical review methodology was adopted in this paper. Grant and Booth [9] argued that a critical literature review explores and evaluates the diverse body of previous research to take stock of what is significant which may result in a model or hypothesis building. Also, Pare´ et al. [10] argued that one strength of critical reviews is their ability to highlight problems and gaps in research about a topic with the aim of strengthening knowledge development by giving a focus and direction for studies or to improve practice. Scholars have noted that despite the relevance of critical reviews, it has “no formal requirement to present methods of the search, synthesis, and analysis explicitly” as done in systematic reviews, and that “the interpretative elements are necessarily subjective and the resulting product is the starting point for further evaluation” [9, p. 97; 10]. Thus, we adopted a critical stance in this paper by engaging with the literature to argue that achieving universal healthcare access by 2030 depends on taking note of the issues we discuss into consideration in nursing care practices, health policy, education, and management.

Despite the limitations of a critical review methodology, some scientific literature review principles were implemented in this study. The paper originated from the first author’s doctoral comprehensive exams organized in February 2021. Included articles were recommended by four expert interdisciplinary doctoral committee members including the second author. Each committee member was asked to recommend at least ten relevant articles on the topic. The objective of the written exam was to discuss the relevance of patient rights and the right to health in advancing the global discourse on universal healthcare coverage (UHC) as crucial to achieving the health sustainable development goal. Recommendations were made by the committee members after a follow-up oral exam. The first author then conducted a further literature search using keywords such as *patient rights, the right to health, universal healthcare coverage, SDG3, patient-centered care, patient-centered communication,* and *nurse-patient communication*. CINAHL, PubMed, Medline, and Google Scholar databases were searched.

The aim of conducting a further literature search was to broaden the discussion to include the significance of patient-centered care practices in achieving universal healthcare access. All included studies in this critical review were empirical research that investigated the healthcare issues around the keyword in different care settings published in English and open access. All relevant articles were read, and findings relevant to our review question were identified, organized, and discussed in this paper.

## Main text

### Universal health coverage as a human rights issue

The right to health and respect for patient rights in healthcare practices are crucial to achieving health for all and universal healthcare access. Particularly, human rights principles of equity, respect for human dignity, non-discrimination, participation, and transparency in healthcare practice and management, health policy design, and implementation are significant to ensuring health for all by 2030 [11]. Human rights and the right to health are advocated for in many international human rights documents. For instance, Article 25(1) of the 1948 United Nations (UN) Universal Declaration of Human Rights (UDHR) and Articles 2.2 and 12 of the 1966 International Covenant on Economic, Social, and Cultural Rights (ICESCR) have all underscored health as a human right [11, 12].

Furthermore, in nursing care, the revised *Code of Ethics for Nurses* observes that "inherent in nursing is a respect for human rights, including cultural rights, the right to life and choice, to dignity and to be treated with respect" [13, p. 2]. It further states that the enjoyment of these rights must not be restricted by a person's "age, colour, creed, culture, disability or illness, gender, sexual orientation, nationality, political [affiliation], race or social status" [13, p. 2]. And the significance of human rights in achieving UHC is further emphasized; “nurses value health care as a human right, affirming the right to universal access to health care for all” [13, p. 18]. This assertion acknowledges the interrelatedness of health, human rights, and other socio-cultural dimensions of being human.

As noted earlier, furthering an SDH approach in UHC implementation will require examining the impact of culture and contextual factors on access to health and healthcare service utilization. In particular, care providers' respect for cultural diversity in care and displaying cultural competency and sensitivity to care can promote access [12, 13]. Therefore, UHC as a human rights issue is essential, which we addressed further in the next section, under the right to health and patients' rights in the context of health for all by 2030.

### Universal health coverage, the right to health, and patients' rights

The right to health is a global concern espoused in several international human rights norms, laws, protocols, and instruments, as indicated in the previous section. The right to health constitutes freedoms and entitlements and recognizes the impact of socio-cultural factors on health. Nonetheless, the right to health does not mean the right to be healthy, given that individual biological and socio-economic conditions differ [12]. The right to health is an inclusive right and privileges non-discrimination and human dignifying practices in healthcare access. It upholds crucial human rights principles, including respect for human dignity, informed consent, self-determination, and participation in healthcare decision-making [12–14]. For instance, *General Comment No. 14* enjoins on State parties the responsibility of making healthcare facilities, services, and personnel available and accessible (easy to reach, affordable, informative). Care services must be acceptable (respectful of medical ethics and culturally appropriate) and of high-quality regarding the services provided, staff, medicines, and facilities/equipment [14]. 

Furthermore, patients' rights are human rights principles emanate from healthcare practices and interactions among care providers, patients, and caregivers. In the *European Declaration on the Rights of Patients*, patients' rights are defined as fundamental human rights in health care, aiming to protect patients' dignity and integrity and respect for the patient as a person [15]. Therefore, the right to health and patients' rights are collectively termed 'human rights in patient care' which outlines the core principles of respect for patients' dignity, privacy, informed consent, and self-determination as critical human rights that must be maintained in healthcare practices and interactions [13, 16]. Per these rights, patients and their caregivers must be fully involved in their care needs, as the ICN *Code of Ethics for Nurses *[13] obligates care providers to provide care services that are person-centered, and respect and value patients' cultural and social characteristics and preferences. Thus, patients and their families must be treated uniquely based on their care needs and circumstances.

For instance, historical antecedents in health research, biomedical experiments, and healthcare practices have shown that communities and specific cultural groups were unethically treated. Moreover, happenings in many modern healthcare institutions are replete with abusive and discriminatory care practices, as cited studies in this and the subsequent sections will show. Hence, the right to health and patients' rights have become critical human rights issues in several countries. National patients' rights charter, service pledges, codes of ethics, and standards of practices for various healthcare professional groups are being developed to safeguard patients' rights. Examples of these documents include the *Helsinki Declaration* for medical research [17], the ICN *Code of Ethics for Nurses*, [13] the *International Code of Ethics for Midwives*, [18] and national patients' charters such as the *Patients' Charter of Ghana*, [19] the *Charter of Patients' Rights in Egypt*, [20] and patient charters of several other countries [21].

These national patients' rights charters have stipulated specific rights and responsibilities of patients that healthcare professionals must uphold and preserve in their care practices and the care process.

Patients' rights are essentially part of the right to health, which, when respected and upheld, can significantly contribute to UHC achievement. First, UHC advocates making healthcare accessible and affordable for all, however, healthcare practices that do not respect patients' rights could lead to inadequate care outcomes and care satisfaction [22]. Moreover, substandard and undignifying patient-provider interactions could prevent future access and utilization of healthcare services and facilities, despite these being available, as observed by Ojwang [23] and Ojwang et al. [24] in studies on patients' rights in Kenyan hospitals. These studies found that, although some patients were treated with dignity and respect, many others were often ignored, discriminated against, intimidated, grouped, and labeled based on their social status. Ojwang et al. [24] argued that these acts by nurses threatened patients' rights to dignity and humanness and often led to patients complaining, criticizing, protesting, and accusing nurses, as ways to assert their rights. These incidents can affect care quality and uptake, especially among vulnerable patients.

The value of patients' rights and effective patient-provider communication and interaction is crucial to achieving UHC. Following this argument, the critical questions to ask are how can effective nurse-patient communication and patient-centered care practices contribute to UHC? How can a study on nurse-patient communication and patients' rights contribute to the UHC discourse's advancement?

### Universal healthcare coverage, healthcare systems, and patients' rights

We acknowledge that advocating a human-rights-based approach to UHC would have implications for healthcare policy implementation and care practices. First, achieving UHC means making healthcare services in all healthcare systems, including traditional medical systems (traditional complementary and alternative medical systems), available, accessible, affordable, and high quality. The traditional medical systems provide healthcare to over 60% of global populations in developing and developed countries. Hence, nation-states and their governments will need to embrace integrative healthcare systems and incorporate traditional medical systems practices and services into their national healthcare policies, planning, financing, and monitoring/evaluation practices [25]. The WHO has noticed that existing barriers, such as geographical and institutional barriers, “the predominance of curative, hospital-based, and disease-based healthcare services” are responsible for poor access to care services over the years [26, p. 35]. Other studies have shown that differences in belief systems about health, illness, and healing between western biomedical and traditional indigenous practitioners constitute a significant barrier to medical system integrations [26, 27].

Another critical factor is that funding for traditional medicine and services is low as governments and their health ministries have challenges budgeting and planning for health services provided by traditional healers [26]. For instance, Carrie et al. observed that “although the overall cost of delivering traditional medicines may be lower than the price of modern medicine, lack of sufficient funding and coverage to enable access may limit the use of traditional medicine in communities” [27, p. 5]. Despite the challenges in achieving integrative healthcare systems, the WHO [25] argues that accessing qualified traditional and complementary medicine is a positive way to achieve universal health coverage.

Besides, issues of patients' rights, cultural sensitivity, and humility will need a holistic examination within different healthcare systems and practices to avoid the marginalization of specific cultural groups. Some societies, cultural groups, and vulnerable populations have had historical experiences of trauma, injustice, and discrimination with their national healthcare systems and healthcare providers. As a result, many of these cultural groups and populations have distrusted their national healthcare systems and care professionals. Hence, to achieve UHC, the historical experiences, unique circumstances, and medical worldviews of these cultural groups and societies must be considered when designing healthcare policies and practice guidelines to achieve health for all, a position taken strongly by the revised ICN *Code of Ethics for Nurses* [13].

Furthermore, the interpretation and application of patient rights principles in particular cultural contexts, medical systems, and healthcare practices (e.g., spiritual healing) may require critical lenses. Many patients' rights principles appear to honour individuality and individual autonomy. However, healthcare decision-making in many cultural contexts is a collective process, sometimes without engaging the patient. Care providers working with an individualistic understanding of human rights and patient rights (influenced mainly by Western ideals) will have to widen their knowledge of human and patient rights to embrace individual rights within collective values. Thus, the compatibility of cultural values, beliefs, and perspectives with Western notions of human rights and patient rights may need a rethink. Healthcare policymakers and care professionals need to reflect on the challenges in implementing UHC policies when collective values and norms overshadow individual rights in positive ways.

### UHC, effective nurse-patient communication, and patient-centered care practices

Patient-centered care (PCC) is an approach to care and interpersonal interaction between care providers and patients during care. It embodies providing care services that respect and respond to patients' unique care needs and preferences [27]. In PCC, patients' experiences, stories, and knowledge must be valued and considered, and patients must engage in the care process as collaborators [27, 28]. The first element of the *ICN Code of Ethics* for nurses––nurses and people––emphasized PCC in nursing care [13]. It focuses on nurses' interactions with people who access care in the healthcare setting and requires that nurses promote a conducive environment in which the human rights, values, customs, and spiritual beliefs of individuals, families, and communities are respected [13, 29, 30].

The nurse-patient dyad is crucial because nurses constitute a strong healthcare workforce and have more contact hours with patients [13]. They carry out various care functions, including recording patients' history and vital statistics, caring for patients' hygiene needs (e.g., bathing/washing and dressing patients), and maintaining ward hygiene [31]. Also, nurses perform some health administrative functions, such as logging cases, and completing forms for patient transfer or health insurance coverage [31, 32].

As nurses continue to engage in medical teams and assume new roles with increasing expertise, their social positions and power dynamics also continue to evolve in the clinical space [33, 34]. This emerging reality makes the social relationship between nurses and patients very complex across different care settings [33]. Due to patients’ varying healthcare needs, their dependence on health professionals for care and support, and the knowledge position nurses occupy, there can be a power imbalance in clinical interactions, mostly in favour of nurses. For instance, in a case study on patient involvement in routine palliative clinical care situations, Glasdam et al. [34] observed that nurses did not readily take up patients’ invitation to dialogue or the knowledge they provide about their body state. The authors argued that the “distribution of power and knowledge between patients and nurses condition nurses to set the agenda and assess when and at what level it is relevant to take up patients’ invitations to involve them in their own case” [35, p. 1618]. According to Glasdam et al., “nurses have the power to define when and how the involvement of patients is going to happen in clinical routine care situations in nursing practice.” ^[35, p. 1624]^ Despite the findings by these scholars, we think that different care situations, patients’ contextual variables/backgrounds, and knowledge state could influence the power dimensions in nurse-patient clinical interactions. For details on power dynamics in nurse-patient interactions, see Glasdam et al. [34] and Mattar e Silva et al. [35] on physician-nurse power relationships.

Moreover, traditionally paternalizing perceptions about patients as passive participants in care routines are changing as patient empowerment principles are advocated in healthcare ethics and practice [36]. For instance, Delaney [36] noted that healthcare organizations and professionals have become more receptive to patient engagement in Australia with the knowledge that patient involvement in their care can enhance safety and care quality. Moreover, the ICN [37], in its position statement on informed patients, expects nurses, patients, and the public to get involved in research that explores the nature and quality of patient information and how it affects health outcomes and nursing care. Thus, nurses have responsibilities of respecting patients' unique values, needs, beliefs, and rights; disseminating appropriate information for effective decision-making; protecting patients' and caregivers' confidentiality. In addition, nurses must demonstrate professionalism, including "respectfulness, responsiveness, compassion, trustworthiness, and integrity" while providing care, as emphasized in empirical studies and the ICN code of ethics [13, p. 2; 38]. The dynamism in nurse-patient communication and interaction makes it an imperative and fruitful ground for engaging in discourses of UHC and patients' rights.

Another essential issue in patient-provider interactions that borders on patient rights is patient autonomy in clinical interactions. As a reviewer rightly observed, patients’ expectation of their degree of autonomy could be compromised when traditional models of bioethics that socialize and position the patient as a passive participant in medical interactions are invoked. Hence, to encourage patient freedom in care, the ICN [39] position statement on nurses and human rights obligates nurses, and by extension, other care providers, to respect patients’ health rights at all times and in all places, including how they express their care needs, worries, and wishes. Nonetheless, several factors can affect patients' expectation of autonomy, including their health status, socio-cultural backgrounds, knowledge of their illness, and previous experiences with healthcare institutions and care providers. Furthermore, notions of “good and bad patient,” as observed by Campbell et al. [40], can influence patients’ expectations of their autonomy in clinical interactions.

Generally, however, studies have shown that when care is patient-centered and human dignity preserving, patient disclosure increases, length of stay in hospitals reduces, and patients become empowered to take care of their health [24, 38]. Moreover, PCC fosters patients' and caregivers' active participation in healthcare decision-making to improve care quality, [41] an essential UHC component. PCC, as a patients' rights practice in care, reduces negative attitudes and personal behaviours from both care providers and patients, thereby reducing conflicts, discrimination, abuse, and disrespect, and increases the uptake of care services and positive nurse-patient relationships [32, 42].

Communication in nurse-patient clinical interactions is effective if patients are (i) provided the needed information about their health conditions, (ii) engaged in the care processes as significant dialogic partners, (iii) listened to, or given the option to choose from alternative care plans and procedures [38, 41]. Effective nurse-patient communication is therapeutic and forms an essential component of PCC. Therefore, the ICN position statement on cultural and linguistic competence recommends that nurses must communicate verbally and in writing in ways sensitive to patients' needs and in a language that patients can understand, if possible, through the use of trained interpreters and translators [43]. Moreover, effective communication in the nurse-patient dyad can reduce misunderstandings between care providers and patients. For instance, Crawford et al. [44] maintain that providing equitable and quality health care is compromised when communication difficulties are present, even when healthcare is accessible.

Care and clinical communication become patient-centered when nurses value patients' beliefs, respect patients' cultural backgrounds, and provide care according to patients' needs, preferences, and unique conditions [13]. It also entails communicating in ways that engage patients and their caretakers and actively listening to patients' concerns [13, 37, 39]. Patient-centered care practices will curtail patient stigmatization and discrimination, especially against people living with HIV/AIDS, mental health illnesses, and the poor, illiterate, and vulnerable patients [22, 32]. Besides, effective nurse-patient communication can increase access to healthcare, patient disclosure, patients' participation in healthcare decision making, positive satisfaction of care outcomes, and accurate healthcare data, essential in promoting UHC [32].

### A Note for consideration in health policy and decision-making

Universal health coverage and providing health for all have been a global agenda for decades and captured as the right to health in most international human rights norms and instruments, as highlighted in this paper. However, improving access to healthcare and ensuring better health for the world's poor, marginalized, and vulnerable populations (e.g., women, children, the elderly, people with disabilities, and Indigenous peoples) has never been vigorously pursued than in the twenty-first century. Starting from early 2000, world leaders at the United Nations agreed to eight development goals achievable within the next 15 years, as the 2015 UN Millennium Development Goals (MDGs). Three of these goals: MDG4 (reduce child mortality), MDG5 (reduce maternal mortality), and MDG6 (Combat HIV/AIDS, malaria, and other diseases), were primarily targeted at health and improving healthcare access to the most vulnerable global populations. Significant gains were made in these goals despite several different challenges. For example, governments were not strictly required to provide disaggregated data in monitoring and reporting the MDGs. Instead, they focused on achieving quantified targets without much emphasis on quality, and attention to human rights-based targets in the MDGs was lacking (see [2] for other critiques).

Following the MDGs' challenges, a more progressive, people-centered, and interdependent set of development goals was adopted again in September 2015, as Sustainable Development Goals (SDGs), achievable by 2030. An overarching health goal, the SDG3, has broader targets and indicators than were found in the health MDGs. Although achieving this particular goal and the rest require financial, logistics, and other resources, emphasizing the human rights dimensions and principles in their implementation process is paramount. Without this focus, the SDGs would likely suffer the same faith as the MDGs. Hence, in this section, critical recommendations are outlined for consideration of health policy and decision-making regarding taking a human rights and patients' rights approach to the UHC/SDG3.

First, awareness of patients' rights must be a critical healthcare decision priority in many countries through public health campaigns. Healthcare advocacy campaigns within healthcare institutions must include information on patients' rights and responsibilities. As a significant sector of the health workforce, nurses should verbally inform, explain to, and educate patients with poor health literacy about patient rights. When patients' rights to dignity, informed consent, participation, among others, are preserved during clinical interactions, abuse and discrimination are reduced, and patient disclosure and uptake of healthcare services increase, [36, 37, 45]. Without much attention to this quality aspect of care, access to available healthcare services will be affected. Many women often refuse facility-based delivery or accessing maternity care in hospitals due to abuse, discrimination, and disrespect. Instead, they prefer home delivery attended by traditional birth attendants [22, 42, 46]. Also, abuse of patients' rights and lack of privacy, confidentiality, and respect, coupled with other ethical dilemmas in HIV/AIDs care settings, affected the uptake of HIV/AIDS care services among this patient population [47].

Secondly, patients' rights regulatory schemes must be instituted in healthcare institutions to enforce patient rights charter regulations and the implementation of nursing ethical codes and standards of practice, and to promote equity and environmental justice when accessing healthcare, as emphasized in the ICN [13, 39]. That is, upholding healthcare ethical standards and patients' rights in healthcare practices will significantly reduce abuse, discrimination, marginalization, and improve care outcomes [48].

Advocating and advancing patients’ rights in nursing practice and care must begin from healthcare educational institutions. Nursing educators must incorporate effective nurse-patient communication and patients' rights models as core components of nursing curricula for knowledge acquisition [13]. Thus, effective nurse-patient communication is crucial to promoting patient-centered care and patient rights in clinical interactions.

Also, healthcare managers can elevate patient rights responsibilities among care professionals by providing regular in-service training on approaches to preserve patients' rights and implement effective communication in practice [28, 37, 48–50]. These in-service training will bolster care professionals' quality, which is critical to achieving UHC [8] and a right to health obligation healthcare institutions must fulfil [12, 14, 39]. Nursing managers should continue to improve nurses' expertise, skills, cultural competence, and quality of care [13, 32, 51]. The active engagement of care managers in the nurse-patient dyad through monitoring and evaluation practices will further support better nurse-patient relationships, patient-centered care practices, and address nurses' personal and professional challenges [36, 52]. For instance, Delaney [36] argues that traditional healthcare management styles focused on illness, intervention, and medication have limitations on optimizing health. Adjusting and aligning clinical care management that honour patients' needs and input have positive outcomes. Moreover, adopting transformational leadership, collaborative and innovative practices can support patients’ participation in, understanding of, and recovery from ill-health. The 21^st^-century nurse faces many demands and challenges, including increasing patient needs, high patient turnout, added administrative duties (e.g., filling diverse forms), and limited time to interact with patients. As a result, there should be an ongoing dialogue between management level officers on how to support healthcare staff to provide quality and patient-centered care that is ethical and patients' dignity preserving.

## Conclusion

Advocating for UHC is crucial and forms one route to healthcare access. Yet, improving care quality and patient satisfaction constitutes another significant matter that needs attention. Broadening UHC implementation processes to include discourses on effective nurse-patient communication, respecting patients' rights, and promoting the right to health is essential to achieving ‘Health for All’ by 2030. Shifting healthcare policy, education, decision-making, and practice to embody the socio-biology and social determinants of health approaches is a priority. The impact of culture and other contextual factors on healthcare access are significant and deserving in UHC discourse.

Healthcare institutional internal structures, hierarchies, and practices that hamper good nurse-patient communication and patients' rights need critical examination. Nurses and nursing managers have active roles to ensure that care is patient-centered and based on human rights, patients' rights, and the right to health principles. Without incorporating a human rights lens and approaches to the implementations of the SDG3, the targets and indicators may be inadequate to protect the poor and vulnerable populations in the global effort to achieve UHC access by 2030.

A limitation of this study, which generally applies to critical reviews is its methodology, which puts less emphasis on literature search and analysis criteria. Thus, the outcome of such a review might be interpreted by some scholars as less scientific. Nonetheless, our critical engagement with the literature and the recommendations made by the expert doctoral committee on both the included studies and an earlier version of the paper will overcome this limitation.

## Data Availability

Data sharing does not apply to this article as no datasets were generated or analyzed during the current study.

## Abbreviations

AIDS: Acquire Immune Deficiency Syndrome
CESCR: Committee on Economic, Social, and Cultural Rights
HIV: Human Immunodeficiency Virus
ICESCR: International Covenant on Economic, Social and Cultural Rights
ICN: International Council of Nurses
MDG(s): Millennium Development Goal(s)
NCD: Non-communicable diseases
PCC: Patient-centered care
PHC: Primary health care
SDGs: Sustainable Development Goals
SDG3: Sustainable Development Goal 3
SDH: Social determinants of health
TB: Tuberculosis
UDHR: Universal Declaration of Human Rights
UHC: Universal health coverage
UNICEF: United Nations International Children Emergency Fund
WHO: World Health Organization
